# The Treatment of Varicose Veins

**Published:** 1897-06

**Authors:** John O’Conor

**Affiliations:** Senior Medical Officer, British Hospital, Buenos Ayres


### THE TREATMENT OF VARICOSE VEINS.

By JOHN O'CONOR, M. A., M. D.

Senior Medical Officer, British Hospital, Buenos Ayres.

After ten years' operative experience I have come to the conclusion that nothing short of total extirpation of the diseased portion of vein merits the term radical.

In numerous instances I have performed the orthodox operation—*i. e.*, removal of many bits; and I regret to say that in a considerable percentage the cure has by no means been permanent.

On referring to one of our most recent standard works, *An American Text-book of Surgical Science*, I find the treatment of this very common complaint is dismissed in some six lines, and I will take the liberty of quoting the following paragraph: "The radical treatment has for its object the complete obliteration of the vein. Many surgical procedures have been devised for this purpose. Of these methods, multiple ligature, as advocated by Dr. Charles Phelps, who ties the vein in thirty or forty places, and excision of the vein in six, eight, or more places, are the best." It is needless to mention that neither of these operations can be really described as radical, for complete obliteration does not necessarily follow even forty ligations, or the removal of even a dozen pieces.

In the operation that I have practised for the past year, and am about to describe, no potential element exists, for the very simple reason that the offending portion of vessel is removed in its entirety.

The limb having been shaved, and disinfected from Poupart's ligament to the ankle, a two-inch incision is made over the saphenous opening, and the internal saphenous trunk is doubly ligated and divided; if no varicosity is present above the knee, the wound is closed, and dressed at once with iodoform gauze. If the femoral portion is affected, after ligation at the saphenous opening, the vein is dissected up, and its tributaries are seized with pressure forceps and ligated. In nearly all cases, if varices are present above the knee, there are also some below; consequently the incision is prolonged downward directly over the vessel until the lowest limit of the disease is reached; the vein is then tied and divided below. In some cases an eighteen or twenty inch incision is necessary.

If the disease does not extend above the knee, after occluding the saphenous trunk, as described above, an incision is made over the affected portion, a ligature is applied above and below, and the whole mass is removed by dissection. It is surprising how easily and rapidly the latter manœuvre may be carried out. Of course, all branches are caught up with pressure forceps, and when the main channel is removed they are ligated. As frequently the external saphenous vein is also affected, its varicose portion is dealt with in a similar manner.

To any one unaccustomed to a ten or twenty inch incision this plan may appear formidable, yet if the vessel is ligatured above and below the varicose area there is not the slightest danger of emboli or pyæmia, and as for hemorrhage, it is so trivial that it does not deserve mention. In a recent severe case I removed twenty-six inches of the internal saphenous, and certainly the blood loss did not exceed two ounces. I have also employed this method in removing large thrombosed veins occurring in the first few months of pregnancy. In this class above all others it is particularly necessary, before manipulating the diseased portion, to occlude the main vein well above the seat of disease, so that if thrombi are dislodged they can not pass into the general circulation.

The time occupied in executing this method certainly does not exceed that in any of the nibbling processes. As to primary union, I find these long wounds heal just as kindly as the short ones do, and with ordinary surgical cleanliness there is nothing to be feared. The insertion of a strand of iodoform gauze as a drain to every four inches of wound is a useful precaution, for it does away with the risk of any blood collections. Personally, I fear the presence of stagnant blood in wounds more than I do germs. Where the former is, doubtless the latter will gain access. "Bichos" deprived of their favorite medium are much more amenable to the natural forces of repair, but with blood aseptic healing is hopeless. It requires only another partner, a few buried silk sutures, to make up a trio that will play the tune of suppuration to the echo.

No bad results have so far followed this method, and all the patients appear grateful.

